# On the selection of relevant historical demand data for revenue management applied to transportation

**DOI:** 10.1057/s41272-022-00371-0

**Published:** 2022-03-07

**Authors:** Ernst Ahlberg, Irina Mirkina, Alfred Olsson, Christian Söyland, Lars Carlsson

**Affiliations:** 1 Universal Prediction AB, Gothenburg, Sweden and Department of Pharmaceutical Biosciences, Uppsala University, Uppsala, Sweden; 2 Stena Line, Gothenburg, Sweden; 3 Universal Prediction AB, Gothenburg, Sweden and Centre for Reliable Machine Learning, University of London, London, UK

**Keywords:** Revenue management, Pricing, Historical demand, Departure clustering

## Abstract

The success of revenue management models depends to a large extent on the quality of historical data used to forecast future bookings. Several theoretical models and best practices of handing historical data have been developed over the years, that all rely on assumptions about underlying distribution and seasonality in the historical data. In this paper, we describe a novel method that compares the fingerprints of the departure to optimise and selects historical departures without making assumptions on data distribution or seasonality. By evaluating the method at the departure level and using the Nemenyi rank test, we show the method’s application in the ferry transportation business and discuss its advantages.

## Introduction

Revenue Management of perishable assets has been thoroughly studied in the airline setting (Littlewood 2005; Weatherford and Bodily 1992; McGill and van Ryzin 1999; Belobaba 2016; Weatherford 2016). Airlines typically optimise the capacity as the number of seats on an aircraft, and seat prices are controlled through a price ladder consisting of fare classes. Preferably, each fare class should have a distinct and unique price interval, such that price overlap between fare classes is minimised. Traditionally these fare classes have also been fenced, to prevent buy-down from corporate travel customers. With the introduction of low-cost airlines, however, a lot of these fences have been removed and differentiation of capacity is primarily driven by passenger comfort and service on-board the aircraft. Over the years, different methods have been proposed to optimise revenue given this setup, including leg-based optimisation and origin-to-destination optimisation (Belobaba 2016). All these methods rely on the use of historical demand data and the ability to describe that data as statistical distributions of demand by fare class (Weatherford 2016). Inaccurate demand forecasts can have significant impact on revenue (Weatherford and Belobaba 2002), although both under and over forecasting of demand can be beneficial in certain circumstances (Mukhopadhyay et al. 2007) leaving the decision to the revenue manager.

In practice, companies utilize historical data in a given time window for demand forecasting and handle deviations in demand (such as seasonality, time of day, etc.) by normalisation of demand. The normalisation converts the historical data to a year average, estimates the demand distributions, and then uses coefficients to convert back to best match the departure of interest. An alternative method is to try to describe deviations in demand through clustering based on different time parameters, such as time of day, day of week, month of year, etc., and by that separate historical data to better account for deviations in the incoming demand. Systematically identifying and handling outliers is important in these approaches as the presence of outliers may result in skewed distributions (Rennie et al. 2021).

In this paper, we propose a novel method based on the similarity between the departure to optimise and historical departures that handles deviations in demand automatically. The expectation is that the method presented here holds for any application where capacity is sold to customers in advance, similar to the airline setting. In this paper we restrict the use of the proposed method to our application in ferry transportation.

In the ferry transportation business, there can be significant deviations between passenger volumes in the high season and in the low season, for example a ferry that can take 2000 passengers in high season could still sail with less than 50 passengers in low season, which means that the validity of normalisation of demand across seasons should be questioned. In the airline industry, such a drop in volume would trigger a change of aircraft, but with the low degree of standardisation of vessels and ports coupled with the comparatively high costs of moving vessels, such a change is not sustainable in the ferry business. Thus, for most parts of the year, there is no achievable limit on the number of passengers on board to optimise for. The number of passengers, however, is not the only capacity to optimise on a ferry vessel. Ferry transportation capacities usually include car deck, cabins, and passengers, as well as restaurants, bars, shops and on-board entertainment. In this article, we focus on optimisation of vehicle pricing on car deck, specifically for the business to consumer market, denoted travel. The car deck is a shared capacity between travel and freight (rolling cargo transportation), with freight being priced through negotiated rates for given volumes, thus only the travel side is considered for dynamic pricing here.

The remainder of the paper is organised as follows. First, we describe the proposed method in the "Method" section. Data and results of the evaluation are then described in the "Results" section and subsequently elaborated on in the "Discussions" section. The paper is concluded with key findings and future outlook in "Conclusions" section.

## Method

The aim of the method is to calculate the similarity between historical departures and the departure to optimise. At any given time, *t*, prior to departure, the method will utilise information about incoming bookings and cancellations, create a fingerprint for the departure, and compare that fingerprint to the corresponding fingerprints of historical departures on that route.^1^ The hypothesis is that departures with similar booking patterns tend to result in similar outcomes (i.e., booked passenger volumes). No additional information, except that contained in the fingerprints, is needed to describe demand variations in the historical departures to capture events or other impact on the demand, eg seasonality.

The method can be described in three steps performed at *t*:

1. Structuring the data in the history pool;
2. Fingerprint creation for the historical data as well as for the departure to optimise;
3. Calculation of a similarity metric between the fingerprints of the departure to optimise and the historical departures.

### Structuring the data

To allow the creation of a fingerprint for each departure, it is essential to describe the booking process. This can be achieved through a set of interventions at different times prior to departure. For each such intervention, information about booking activity since the last intervention is captured. This can be accomplished either through a set of predefined data collection points (DCPs), or through assessing the total booking distribution versus time to departure in the history pool and selecting $n_{DCP}$ time steps such that each step contains an equal amount of incoming bookings. In addition, a floating DCP can be created at time *t* if *t* does not coincide with a predefined DCP. These procedures result in a sorted list *D* of times to departure to iterate over, where $D = [d_1,\ldots ,d_t,\ldots ,d_{n_{DCP}}]$, that we can use for fingerprint creation.

### Fingerprint creation

The fingerprint for each departure is created as a vector comprised of incoming bookings and cancellations in each interval between DCPs. For each $d \in D$, the number of bookings and cancellations since $d-1$ for each fare class (FC) is calculated and appended to the fingerprint. This fingerprint tends to be sparse and the dimensionality of the fingerprint is $2 \cdot n_D \cdot n_{FC}$. An alternative is to calculate q-bookings (Belobaba and Hopperstad 2004) and q-cancellations for each *d*, generating a dense fingerprint with the dimensionality of $2 \cdot n_D$. Generation of a fingerprint for the departure to optimise requires a reference point. The reference point can be a single departure in the history pool or an average of relevant departures from the history pool. The fingerprint for the departure to optimise is then constructed by using the information from the departure where $d \le t$ and then populating the remainder of the fingerprint, $d > t$, using the reference point.

### Similarity calculation

Comparison between the fingerprint for the departure to optimise and the fingerprints of historical departures can be performed using any similarity or distance metrics, with or without weighting, and can be sorted into a ranked list. Depending on availability of historical data, one can either decide to take the top *n* departures or all departures above or below a given threshold, depending on the similarity or distance metric of choice for further processing and forecasting.

## Results

To demonstrate the method, a retrospective study using real world data has been conducted on two ferry routes. Route *A* has a longer sailing with an early booking pattern and route *B* has a short sailing and tends to be booked closer to departure. Both versions of the fingerprint: using fare classes, *Sparse*, and using q-bookings, *Dense*, have been computed. The *Sparse* fingerprint uses a fixed DCP list and the *Dense* fingerprint uses a dynamic data driven DCP list as described above. The Euclidean norm is used as a distance metric and the 10 closest departures are selected as the history pool for the optimisation. For both fingerprints a weighted Euclidean distance is used that increases the emphasis on the departure at hand compared to the future as described by the reference point. This weighting increases as the time to departure decreases. Since no obtainable physical limit exists for the volume to optimise, the mean of the selected historical departures has been used.

The optimisation uses EMSRc (Hossein and Weatherford 2017) based on pickup (Wickham 1995; L’Heureux 1986) and has been calculated with and without q-forecasting. Thus for the evaluation, four methods have been evaluated together with the base predictor of last years outcome. The methods are denoted *Dense*: *EMSRc*, $Dense:EMSRc\_QFC$, *Sparse*: *EMSRc*, $Sparse:EMSRc\_QFC$ and *outcome*: *LY*. The results have been divided by the respective departure outcome and subtracted by one, thus showing results as deviations from the respective observed departure outcome. Furthermore, the retrospective analysis is based on 150 departures departed in February 2020, prior to COVID-19, and uses historical data with departure dates starting from 2019-01-01. As a reference point for the departure to optimise, the corresponding departure last year with the same time of day and day of week has been used. The analysis starts at 126 days to departure and has been calculated with a straight weekly updating schedule.

### Departure level results

**Fig. 1:**
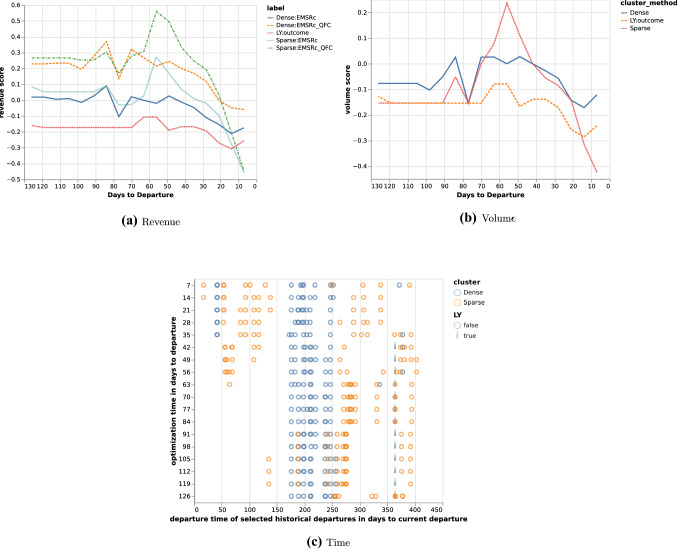
Revenue and volume estimates per method and time of departure for historical departures. Calculated for departure 9037 on route *A*

**Fig. 2:**
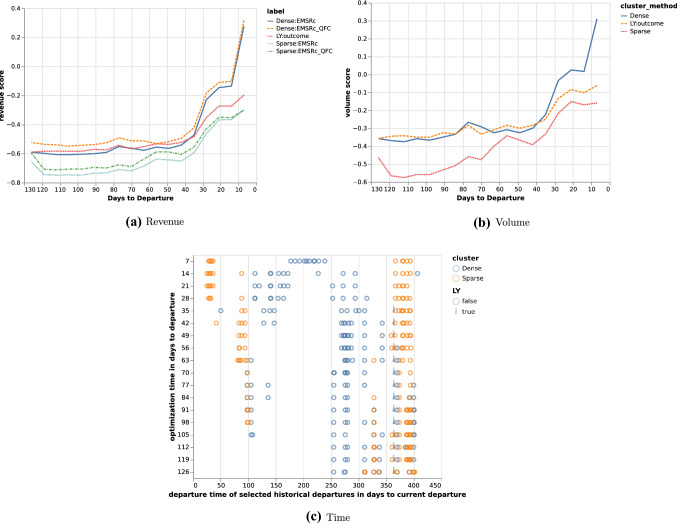
Revenue and volume estimates per method and time of departure for historical departures. Calculated for departure F06C on route *A*

**Fig. 3:**
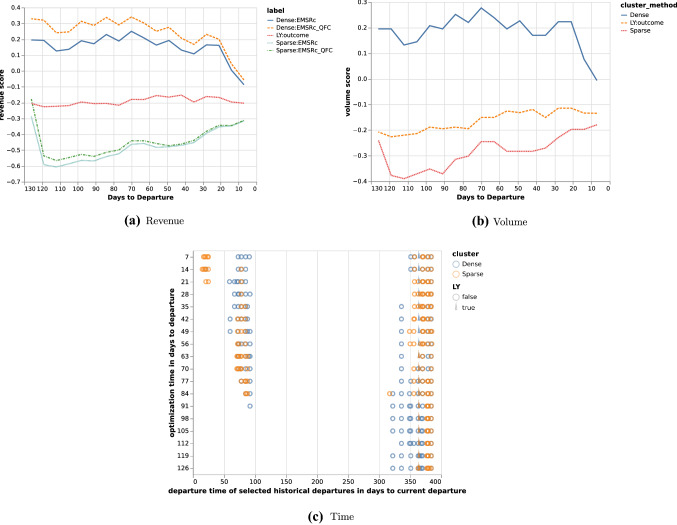
Revenue and volume estimates per method and time of departure for historical departures. Calculated for departure A7AF on route *B*

To evaluate the methods on a departure level, observed and expected volume has been used together with revenue calculated based on volume and fare class, converting all vehicles to the predominant vehicle type such that numbers are directly comparable. For each departure, revenue and volume has been plotted versus days to departure as well as the departure dates of the history pool described as days to current departure. Three departures are shown in the paper in Figs. 1, 2 and 3, where subfigure *a* shows the estimated revenue for the different methods and subfigure *b* shows the estimated volumes calculated at each optimisation point. Subfigure *c* shows the optimisation points in days to departure on the *y* axis and the time between the current departure and the departure date for the selected historical departures on the *x* axis. Thus subfigure *c* shows how the selected historical departures are spread throughout the year. To complete the results, relative volumes per fare class are plotted for the departures from the history pool at each optimisation point together with the expected volume and fare class distribution by the methods and the departure outcome. In the paper, only a subset of the optimisation points are shown for the departures in Tables 1, 2 and 3. Each histogram shows current bookings in blue, future bookings in red, current cancellations in orange and future cancellations in green. For each optimisation point, the top row of figures corresponds to selections using the *Dense* fingerprint and the bottom row corresponds to the *Sparse* fingerprint. The first column corresponds to the *EMSRc* optimisation method, the second to the $EMSRc\_QFC$ optimisation method and the third to the departure outcome. The remaining columns correspond to the selected departures from the history pool. The focus of the analysis is on distribution shape and color, showing how a given departure and its optimisation changes over time.

**Table 1:**
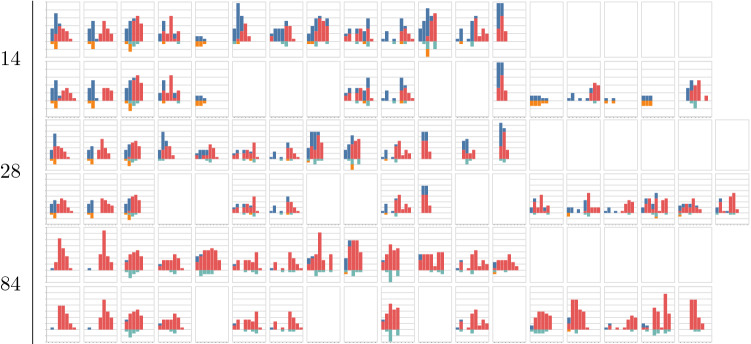
Selected historical departures, optimised allocations and outcome for selected optimisation point in days to departure for departure 9037 on route *A*

**Table 2:**
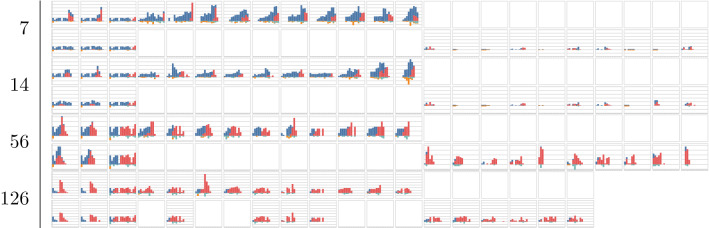
Selected historical departures, optimised allocations and outcome for selected optimisation points in days to departure for departure F06C on route *A*

**Table 3:**
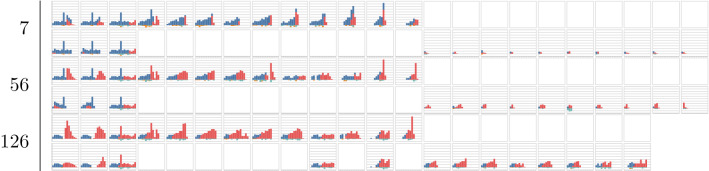
Selected historical departures, optimised allocations and outcome for selected optimisation points in days to departure for departure A7AF on route *B*

### Nemenyi test results

**Fig. 4:**
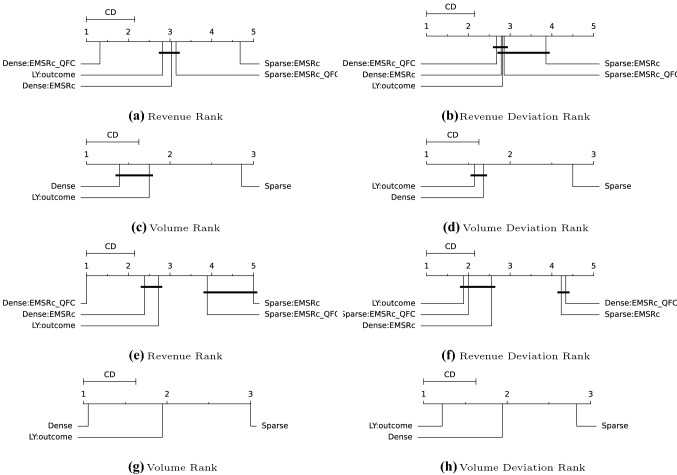
Nemenyi tests for route *A*. For subfigures **a**–**d** ranks are compared on departure level and for subfigures **e**–**h** ranks are compared based on optimisation point

**Fig. 5:**
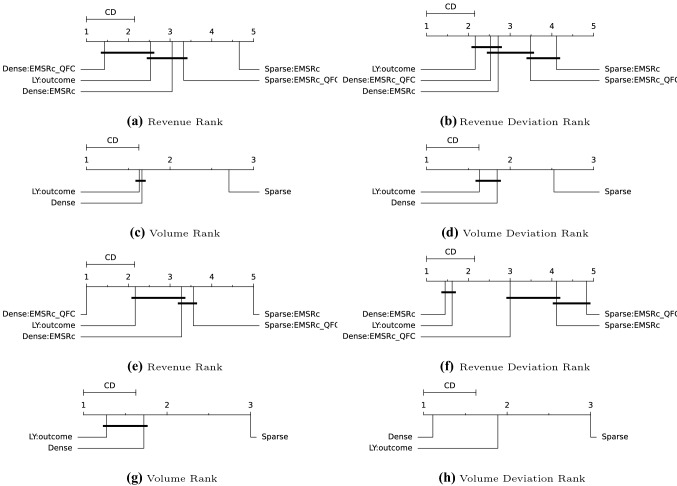
Nemenyi tests for route *B*. For subfigures **a**–**d** ranks are compared on departure level and for subfigures **e**–**h** ranks are compared based on optimisation point

The Nemenyi rank test has been used to compare the methods on an aggregate level (Nemenyi 1963). Figures 4 and 5 compare the ranks of the different methods with respect to volume and revenue as well as their respective deviations from the obtained outcome. The data has been grouped in two different ways for the calculations: by departure and by optimisation point, as days to departure. For revenue and volume rank, the highest number has the highest rank and for the deviation from the outcome the lowest number has the highest rank. The interpretation of the Nemenyi test is that given an evaluation metric, methods which average ranks across a set of evaluations are separated by more than the critical distance, CD, can be said to be different. As an example, Fig. 4c compares expected volume from three predictors, *Dense*, *Sparse* and last years outcome. The *Dense* method has the highest rank, *i.e.* on average it gives the highest volume estimate. It can however not be separated from last years outcome since the ranks of the methods are closer than the critical distance. The *Sparse* method however gives the lowest volume estimate on average and it is significantly different from the two other methods.

## Discussion

The proposed fingerprints, *Dense* and *Sparse*, provide a way to select relevant historical departures for revenue management. Using a fingerprint and a similarity metric makes it possible to estimate demand at different fare classes without:

1. making assumptions about the underlying distributions;
2. segmenting the historical departures into subcategories such as seasonality.

Figure 1c shows that similar departures were available roughly three months before departure in this example. This flexibility allows for efficient usage of the historical data, regardless of when it is generated and can be seen for all three departure examples in Figs. 1, 2 and 3. Furthermore the results show that, in the current setup, the dense fingerprint tends to generate higher volumes compared to the sparse fingerprint, as can be seen in Figs. 4 and 5c and g. That is also visible in Table 1 showing that the *Sparse* fingerprint has identified departures with lower over-all volumes as more similar. One reason for this might lie in the fingerprint itself, in essence it might be too sparse, resulting in close to zero distances for most departures. If the historical departures have not sold in the same fare classes at the same time prior to departure a lot of the potential similarity between departures is lost, thus the *Sparse* fingerprint is more dependent on a stable RM steering strategy. A second effect for the *Sparse* fingerprint is that closer to departure, it tends to identify departures with low future demand. This might be an effect of the applied weighting scheme, not putting enough weight on the potential future bookings. The *Dense* fingerprint handles some of these shortcomings by recalculating demand in each time span into q-demand. Figure 1b shows that although other departures are taken into account, it tends to depend on last years outcome, which is to be expected since that is the reference point for the fingerprint. In comparison to last years outcome and grouping by departure, the *Dense* fingerprint is closer than the *Sparse* fingerprint as can be seen in Figs. 4 and 5c and d. The deviation in subfigure *d* shows that the *Dense* fingerprint overestimates the demand. However, looking at Figs. 4 and 5g and h, where ranks are compared by optimisation point rather than by departure, show that the two routes have been steered differently. Route *A* have been steered in line with last years outcome while route *B* has been steered by the *Dense* method. During February 2020, the methods described here were available as recommendations to the trade optimisers, and could be manually applied.

Turning the focus to the revenue evaluations, Figs. 4 and 5a and e show that $EMSRc\_QFC$ consistently provides higher revenue estimates compared to *EMSRc*, regardless of the fingerprint used. Although this is expected, and not the primary focus of the study, it is still reassuring to see. Looking at the comparison to the observed revenue in Figs. 4 and 5b and f, it is not possible to separate the optimisation methods using the *Dense* fingerprint and last years outcome, since the critical distance is too high. Figs. 4 and 5f also indicate that route *A* was primarily steered by the last year reference and route *B* using *Dense*: *EMSRc*, although the results are too close to call.

Figure 2 and Table 2 show another departure from route *A*. The results using the *Dense* clustering follow the last year outcome curve until 28 days before departure where it starts to pick up a set of newer historical departures. At 7 days before departure the updated fingerprint leads to a significant shift in history selection, increasing expected volume and revenue, but not increasing fare class. Thus given that the increased volume materialises, the revenue should be higher. Comparing selected historical departures in Table 2, 7 and 14 days prior to departure, as well as Fig. 2c show a clear difference. Also, at 28 days to departure the *Dense* fingerprint has no longer identified last years departure as one of the 10 departures to compare to. This can be seen in Fig. 2c where last years departure is marked with a wedge. In such a clear case as this, one could argue that it would have been beneficial to pick up the difference between the current departure and its last year reference earlier, but that is not part of this study. Using the same departure, the optimisation methods indicated early that there was demand in higher fare classes and that the price could be increased. Close before departure, the optimisation wanted to open down, to stimulate volume. If the additional expected volume materialises then it would increase total revenue, otherwise it would lead to a lower total revenue since the customers would buy the tickets cheaper.

Figure 3 and Table 3 show a departure from route *B* where the last year reference under estimates the demand and the *Dense* fingerprint method over estimates it. Much like the second example, the optimisation methods indicate demand in higher fare classes early on. In the end, the volume and revenue converges to the departure outcome. In a retrospective study it is not possible to truly evaluate the impact of any method since the data generating process is affected by the decisions taken in the optimisation. To perform such a comparison, it is required to perform a controlled experiment, and unfortunately it needs to be tested in a live environment. Devising a test strategy that allows for this kind of testing whilst minimising risk is a key piece of future work.

The method has however been rolled out internally, first for manual approval by revenue managers and then automated. The roll out is not a controlled study but early indications point towards a 10% increase in revenue for the recommendation process and more once put into automation.

As have been demonstrated, the selection of reference point is important for the performance of the method. Indicative studies show that the ability to update the reference point if the departure to optimise deviates significantly from the reference point will have a positive impact on the outcome of the optimisation. Here the fingerprint can be used again, first to compare the fingerprint of the reference point up to *t* with the fingerprint for the departure to optimise, and then to identify and suggest new reference points from the historical data that match the fingerprint up to *t*. Performing such a selection automatically comes with a risk similar to the one seen for the *Sparse* fingerprint, when the reference to future bookings are removed, a departure with a high similarity up to *t*, can still deviate in future demand. One reason for this is inherent in the booking process where the number of bookings increase closer to departure. We leave this topic for further research.

## Conclusions

This paper proposes a novel method that describes the booking process of a departure as a fingerprint that can be compared to historical departures for selection of reference departures for revenue management. The evaluation shows that this creates a flexible method that can take advantage of departures with similar fingerprints regardless of when such departures occur, thus it can handle both rare events, eg outliers, and seasonality. This allows optimisation of future departures without the need to make assumptions of underlying distributions or to compute seasonality through normalisation of historical departures. The method shows great potential for both practical and theoretical advancements in revenue management in transportation. There are three key topics for future research; methods for optimal selection of reference point(s) and corresponding updating strategies, risk limited testing strategies for live evaluation of revenue management steering strategies and further development of fingerprint parameterization.

## Footnotes

1 Throughout the paper, we use terminology adopted in the ferry transportation industry. ‘Route’ here has the same meaning as ’the departure leg’ in the airline industry
